# Pancake Syndrome (Oral Mite Anaphylaxis)

**DOI:** 10.1186/1939-4551-2-5-91

**Published:** 2009-05-15

**Authors:** Mario Sánchez-Borges, Raúl Suárez-Chacon, Arnaldo Capriles-Hulett, Fernan Caballero-Fonseca, Victor Iraola, Enrique Fernández-Caldas

**Affiliations:** 1 Centro Médico-Docente La Trinidad, Caracas, Venezuela; 2 Clínica El Avila, Caracas, Venezuela; 3 Policlínica Metropolitana, Caracas, Venezuela; 4 Hospital San Juan de Dios, Caracas, Venezuela; 5 Centro Médico de Caracas, Caracas, Venezuela; 6 Laboratorios Leti, Madrid, Spain; 7 Allergy Innovations GMBH, Starnberg, Germany

**Keywords:** anaphylaxis, exercise-induced anaphylaxis, food allergy, immunoglobulin E, mites

## Abstract

Oral mite anaphylaxis is a new syndrome characterized by severe allergic manifestations occurring in atopic patients shortly after the intake of foods made with mite-contaminated wheat flour. This clinical entity, observed more frequently in tropical/subtropical environments, is more often triggered by pancakes and for that reason it has been designated "pancake syndrome". Because cooked foods are able to induce the symptoms, it has been proposed that thermoresistant allergens are involved in its production. A novel variety of this syndrome occurs during physical exercise and therefore has been named dust mite ingestion-associated exercise-induced anaphylaxis. To prevent mite proliferation and the production of anaphylaxis, it has been recommended that wheat flour be stored at low temperatures in the refrigerator.

## Introduction

Domestic mites have been recognized as the most important allergen source responsible for respiratory allergic diseases, such as rhinitis and asthma, of high prevalence worldwide [1]. Since 1982 we have studied a group of atopic patients who developed severe allergic symptoms immediately after eating foods prepared with wheat flour contaminated with various species of mites. This syndrome was designated as oral mite anaphylaxis (OMA) or "pancake syndrome".

The present article summarizes the observations made by our group and other investigators on this new allergic condition. The first reports on oral anaphylaxis from mite ingestion in the United States came from Detroit, Mich, and Philadelphia, Pa [2,3].

Previously, other clinical manifestations associated with the intake of mite-contaminated foods had been published. In 1963 Herranz reported the case of a 56-year-old female patient who died after a massive ingestion of mites contained in a pap made with milk and wheat flour. Autopsy revealed intense intestinal irritation, disseminated granulomas in multiple organs, and the presence of many mites of the species *Tyroglyphus farinae *(*Acarus siro*) in stools and gut [4]. Hinman and Kampmeier had described 2 patients with diarrhea due to "intestinal acariasias" produced by infestation with *Tyroglyphus longior Gervais*, a member of the family Tyroglyphidae, arthropods frequently found in dried fruits, dried seeds, grains, cereals, and cheese [5].

Various elements with pathogenic potential, including allergenic contaminants, may be present in the food. They include microorganisms (bacteria, viruses, fungi, protozoa, and helminths), toxins, chemicals, food additives (dyes and preservatives), food allergens, allergens from other contaminating foods, cross-reactive allergens from pollens or latex, drugs (especially penicillin), arthropods (mites), and insects (cockroaches).

## Oral Anaphylaxis from Mite Ingestion

There are reports from various countries, including the United States, Japan, Brazil, Taiwan, Spain, and Venezuela, where OMA has been observed (Table 1) [2,3,6-13]. Unpublished cases have also been reported in the Dominican Republic and Israel. Only 2 studies from the Canary Islands and Venezuela have presented small series of cases [8,9].

**Table 1:** Publications on Oral Mite Anaphylaxis

Author (year)	n	Age	Sex	Foods (Location)	Mites	NSAID Hypersensitivity
Erben et al (1993)[2]	1	48	M	Beignets (Detroit)	*D. farinae*	--
Spiegel et al (1994)[3]	1	17	F	Beignets (Philadelphia)	*D. farinae*	--
Skoda-Smith et al (1996)[6]	1	14	M	Pizza dough (Birmingham, Ala)	*D. farinae*	Familiar
Matsumoto et al (1996)[7]	2	11	M1	Okonomi-yaki (Kumamoto, Japan)	*T. putrescentiae*	--
		14	F1			
Blanco et al (1997)[8]	16	13-38	M4	Various (Canary Islands, Spain)	*D. farinae, T. entomophagus*	87%
			F12			
Sánchez-Borges et al (1997)[9]	30	13-45	M14	Various (Caracas, Venezuela)	*D. farinae, Suidasia *spp., *A. ovatus*	66.6%
			F16			
Guerra Bernd et al (2001)[10]	1	18	F	Polenta (Porto Alegre, Brazil)	*Tyrophagus, D. pteronyssinus, D. farinae*	Yes
DeMerrell et al (2004)[11]	1	11	M	Beignets (New Orleans, La)	*D. pteronyssinus*	--
Wen et al (2005)[12]	1	8	M	Pancakes (Taipei, Taiwan)	*B. freemani*	--
Hannaway and Miller (2008)[13]	1	52	F	Pancakes (Massachusetts)	*D. farinae*	--

In general, there is a predominance of reports from countries located in the intertropical area, probably because in those regions there are environmental conditions favorable for mite reproduction, especially higher temperature and relative humidity, for longer periods of time.

Patients described in Detroit, Mich, and Philadelphia, Pa, had anaphylaxis after eating beignets made with flour delivered by mail from New Orleans,[2,3] whereas only 2 cases were observed out of the tropics, one in Porto Alegre, Brazil, and the other one in Massachusetts [10,13]. Many other patients with OMA are not diagnosed because of the lack of appropriate clinical inquiry or the lack of referral to the allergologist for proper study and management. This explains why some patients can develop the clinical picture repeatedly until the correct diagnosis is made.

## Clinical Picture

Table 2 presents the clinical data obtained from our first 30 patients [9]. The majority of subjects are adolescents and young adults although OMA can occur in children,[14] without a predominance of sex, generally with a previous history of rhinitis, asthma, and/or atopic dermatitis, who developed their symptoms between 10 and 240 minutes after receiving the food.

**Table 2:** Clinical Data on 30 Patients Who Presented Oral Mite Anaphylaxis

**Patient No**.	Sex	Age (y)	Reaction Time (minutes)	Previous Allergic History	Foods
1	M	20	60	Asthma, rhinitis	Pancakes
2	M	18	NA	Rhinitis	Pancakes
3	M	26	30	Asthma, rhinitis	Pancakes
4	M	24	10	Rhinitis, conjunctivitis	Pancakes, Pizza
5	M	16	20	Asthma, rhinitis	Sponge cake
6	F	21	NA	Asthma, rhinitis	Sponge cake, pasta
7	M	24	30	Asthma, rhinitis	Sponge cake
8	F	18	NA	Asthma, rhinitis	Sponge cake
9	F	24	NA	Asthma, rhinitis	Pasta
10	F	25	20	Rhinitis	Pancakes
11	F	19	30	Rhinitis	Pancakes
12	F	15	15	Rhinitis	Pancakes
13	F	13	60	Rhinitis, atopic dermatitis	Pancakes
14	M	15	NA	Asthma, rhinitis	Pancakes
15	M	14	30	Asthma, rhinitis	Pancakes
16	M	19	30	Rhinitis	Pizza
17	F	27	30	Asthma, rhinitis Atopic dermatitis	Pancakes
18	M	15	60	Asthma, rhinitis	Pancakes
19	M	31	30	Rhinitis	Cornmeal cake
20	M	20	240	Rhinitis	Bread
21	F	39	30	Rhinitis	Pancakes
22	F	39	30	Rhinitis	Cheese/wheat sticks
23	M	16	30	Rhinitis	Parmigiana Steak
24	F	16	90	Asthma, rhinitis	Alfajor
25	F	37	30	Asthma, rhinitis	Parmigiana Steak
26	F	17	30	Asthma, rhinitis	Pancakes
27	F	24	60	Asthma	Pancakes
28	F	45	NA	Rhinitis	Sponge cake
29	F	25	15	Asthma, rhinitis	White sauce
30	M	15	30	Rhinitis	Pancakes

Clinical manifestations are summarized in Table 3, with breathlessness, facial and laryngeal angioedema, wheezing, and other upper and lower respiratory tract symptoms being the most common. The clinical presentation may be very severe, determining the need for hospitalization in the intensive care unit due to laryngeal edema and acute respiratory failure. There are 2 deaths secondary to the ingestion of mite-contaminated foods published in the medical and legal literature [15-17].

**Table 3:** Clinical Manifestations of Oral Mite Anaphylaxis (Pancake Syndrome)

Symptoms	No. Patients (%)
Breathlessness	27 (90)
Angioedema	15 (50)
Wheezing	12 (40)
Rhinorrhea	9 (30)
Cough	8 (26.6)
Stridor	6 (20)
Dysphagia	6 (20)
Urticaria	6 (20)
Abdominal cramps	4 (13.3)
Conjunctivitis	3 (10)
Skin rash	2 (6.6)
Dysphonia	2 (6.6)
Sneezing	1 (3.3)
Vomiting	1 (3.3)
Cyanosis	1 (3.3)
Pruritus	1 (3.3)
Tachycardia	1 (3.3)

Oral allergy syndrome (OAS, pollen-food allergy syndrome) is characterized by an immediate hypersensitivity reaction generally localized to the lips and oral mucosa after contact with crude fruits or vegetables containing allergens that share homology with pollen proteins, the original source of sensitization. Because the responsible allergens are thermolabile proteins, cooked foods are tolerated. OAS is not associated with oral mite anaphylaxis.

## Etiology

Incriminated foods are usually prepared with wheat flour, including pancakes, sponge cakes, pizza, pasta, bread, parmigiana steak (prepared with contaminated grated bread), and white sauce. Pancakes are the foods most frequently involved (53.3% of our patients) and for that reason we proposed in 2001 the designation of "Pancake syndrome" for this clinical entity,[18] a nomenclature recently supported by other investigators [13,17].

Other foods have also been found to induce OMA, including beignets, "okonomi-yaki" (bonito and mackerel covered with flour), cornmeal cakes made with a commercial mix containing wheat and corn flour, and polenta. Other foods that can be contaminated with mites when stored for long periods at room temperature are cheese, ham, chorizo, and salami [19-21].

The observation that symptoms appeared after eating foods cooked by heating led us to perform skin prick tests with contaminated wheat flour unheated and after heating at 100°C for 1 hour in 13 mite-allergic volunteers. A significant reduction of wheal diameters was observed when the tests were done with heated flour; nevertheless, the tests continued to be positive. These results suggest that allergens inducing OMA are thermoresistant.

Different mite species are present in the flours producing OMA. Among them are house dust mites (*Dermatophagoides pteronyssinus *and *Dermatophagoides farinae*), and storage mites such as *Suidasia *spp., *Aleuroglyphus ovatus, Lepidoglyphus destructor, Tyrophagus putrescentiae, Thyreophagus entomophagus, Blomia tropicalis*, and *Blomia freemani*. The issues on allergenic cross-reactivity between different domestic and storage mite species have been recently reviewed by Fernández-Caldas et al [22] and by Arlian et al,[23] and could explain why patients sensitized to domestic mites show systemic reactions when exposed to storage mites by the oral route. However, it must be mentioned that mite species previously regarded as "storage mites" are present in domestic dwellings in Caracas, as recently shown by our group [24].

## Relationship Between Oral Mite Anaphylaxis and Nsaid Hypersensitivity

A high prevalence of cutaneous hypersensitivity (urticaria and angioedema) to aspirin and other nonsteroidal anti-inflammatory drugs (NSAIDs) has been observed in patients who develop OMA [8,9] (see Table 1). In 1997 we proposed a "New Aspirin Triad" characterized by allergic rhinitis, aspirin/NSAID hypersensitivity, and severe reactions to mite-contaminated foodstuffs [25].

The reasons for this association are not known because mite allergy is mediated by a specific IgE response to mite allergens, whereas most patients with urticaria and angioedema from NSAIDs react to various anti-inflammatory drugs of diverse chemical composition ("cross-reactions") that are regarded to be nonallergic hypersensitivity reactions likely mediated by the inhibition of the enzyme cyclooxy-genase-1 of arachidonic acid metabolism, which results in enhanced production of cysteinil-leukotrienes and decreased 26 prostaglandin E_2 _(PGE_2_) synthesis [26].

In collaboration with Canadian investigators we demonstrated that allergenic mite extracts can inhibit cyclooxy-genase-1 in vitro [27]. More recently, in collaboration with Dr. Luis Caraballo and Dr. Nathaly Acevedo (Instituto de Investigaciones Inmunológicas, Universidad de Cartagena, Cartagena, Columbia), we observed an increased frequency of the C allele of *leukotriene C4 synthase *(*LTC4S*), the enzyme responsible for the synthesis of leukotrienes, in cross-reactive NSAID hypersensitive patients with urticaria and/or angioedema. In such study we confirmed our previous results on the increased prevalence of the atopic trait in those patients [28].

Various lines of evidence suggest an interaction between IgE-specific immune responses and the leukotriene pathway. The so-called "atopic" genes implicated in allergic inflammation, including those determining the production of the cytokines IL-4, IL-5, and IL-13, are located in the 5q22-q35 chromosomal region of chromosome 5, close to the *LTC4*S gene, the main locus that regulates cysteinyl leukotriene synthesis in humans. We previously proposed that perhaps this colocalization could explain the association between atopy (or mite allergy) and NSAID hypersensitivity [9].

More recently, we observed that patients cross-reactive to NSAIDs show larger wheals when prick-tested with *B. tropicalis *allergenic extract than atopic non-NSAID-sensitive patients [29]. In this regard it is interesting to mention that Acevedo et al recently described an association of the *A-444C *allele of the *LTC4S *gene and low levels of IgE antibodies to *D. pteronyssinus*, low total IgE, and monosensitization and suggested that *LTC4S *could be involved in the regulation of IgE response to mite allergens [30].

## Cysteinyl Leukotrienes Modulate the Allergic Response

Cysteinyl leukotrienes can enhance IgE and IgG production by human B cells [31]. *LTC4S *knockout mice have markedly reduced antigen-induced TH2 pulmonary inflammation [32]. Also, various studies have shown that IL-4 and IL-13 enhance the number of cysLT1 and cysLT2 receptors on T, B, and antigen-presenting cells [33-35].

There are also some recent clinical and experimental observations that support a modulating role for leukotrienes in allergy. For example, Aihara et al reported that acetylsalicylic acid facilitates food-dependent exercise-induced anaphylaxis,[36] and aspirin may facilitate anaphylaxis to wheat,[37,38] prawns,[39] and peanuts [40] (Table 4).

**Table 4:** Observations Supporting Interrelationships Between Cysteinyl-Leukotrienes and the Atopic Condition

• Excessive production of leukotrienes in patients with hypersensitivity to NSAIDs
• Increased prevalence of atopy (allergy to mites) in patients with hypersensitivity to NSAIDs
• A genetic polymorphism of *LTC_4 _synthase *gene is present in patients with NSAID hypersensitivity
• A genetic polymorphism of *LTC_4 _synthase *gene regulates the production of specific IgE
• "Atopic" and *LTC_4_S *loci are located closely in chromosome 5
• Genetic polymorphisms of leukotriene receptor genes are associated with atopy
• Leukotrienes enhance the production of IgE by human B cells
• IL-4 and IL-13 increase the number of leukotriene receptors
• Acetylsalycilic acid enhances food-induced anaphylaxis and food-dependent exercise-induced anaphylaxis
• Patients with oral mite anaphylaxis show increased prevalence of NSAID hypersensitivity
• Mite allergenic extracts inhibit cyclooxygenase activity in vitro

**Figure 1:**
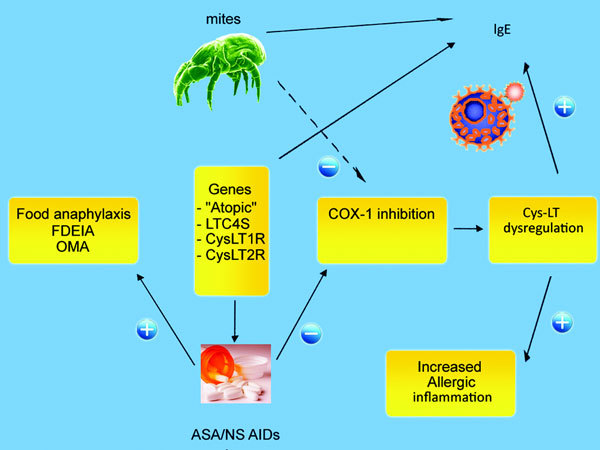
**Interactions between mite allergy and leukotriene-mediated inflammation: (-) inhibition; (+) enhancement**.

Additionally, in experimental animals it has been observed that aspirin increases the permeability of the gastric mucosa to proteins and the development of anaphylaxis [40]. Figure 1 summarizes the possible interrelationships between mite allergy and NSAID hypersensitivity.

## Risk Factors and Diagnosis

The following elements have been identified as factors increasing the risk for OMA: (1) History of atopic disease; (2) sensitization to mites; (3) cutaneous hypersensitivity to NSAIDs (urticaria and angioedema); (4) ingestion of pancakes or other foods prepared with mite-contaminated wheat flour; (5) ingestion of more than 1 mg of mite allergen (more than 500 mites per gram of flour) [9].

The diagnosis is based on the following findings: (1) Compatible symptoms occurring after the intake of foods prepared with wheat flour; (2) previous history of rhinitis, asthma, atopic dermatitis, and/or food allergy; (3) in vivo or in vitro demonstration of IgE-mediated sensitization to mite allergens; (4) positive immediate-type skin test with extract of the suspected flour; (5) negative skin tests to wheat and to uncontaminated flour extract; (6) clinical tolerance of foods made with uncontaminated wheat flour; (7) microscopic identification of mites in the suspected flour; (8) presence of mite allergens in the flour; (9) hypersensitivity to NSAIDs.

## Pancake Syndrome and Exercise-Induced Anaphylaxis

Recently, we observed a 16-year-old girl with previous history of asthma, rhinoconjunctivitis, atopic dermatitis, and allergy to clams, who presented generalized itching, facial angioedema, and breathlessness when playing soccer 30 minutes after eating a lunch consisting exclusively of pancakes. Treatment in the emergency room consisted of intravenous corticosteroids, chlorpheniramine, and nebulized albuterol. After discharge, oral methylprednisolone, desloratadine, inhaled budesonide, and formoterol were given.

Results of skin prick tests done to the patient 2 weeks after the episode are presented in Table 5. These were positive for *D. pteronyssinus, B. tropicalis*, and the extract of the flour used to make the pancakes, whereas they were negative for wheat and other food extracts and for other inhalant allergens. Microscopic analysis of the flour showed the presence of *Suidasia medanensis*, quantified in 4814.8 mites per gram of flour. Der p1 and Der f1 allergens were not detected in the flour.

**Table 5:** Results of Skin Prick Tests in a Patient With Dust Mite Ingestion-Associated Exercise-Induced Anaphylaxis

Allergen	Wheal Diameter (mm)
*D. pteronyssinus*	23
*B. tropicalis*	13
Involved wheat flour extract	6
Wheat	0
Other food extracts (milk, egg, oat, barley, corn, soy, peanut, orange, chicken, pork, beef, cocoa, shellfish mix, fish mix, tomato, salmon, tuna, pineapple, and strawberry)	0
Other inhalant extracts (mold mix, Bermuda grass,* ragweed, ryegrass, tree mix, feathers, dog, cat, and cockroach)	0
Glycerosaline solution	0
Histamine	6

*Do you mean "Bermuda grass" and not just "Bermuda"?

The results of skin tests are in agreement with previous studies that showed a high degree of cross-reactivity between *S. medanensis, B. tropicalis*, and *D. farinae *[42].

This variant of pancake syndrome has been designated as dust mite ingestion-associated exercise-induced anaphylaxis [43]. A similar clinical picture of exercise-induced anaphylaxis related to foods contaminated with the fungus Penicillium lanoso-ceruleum was previously reported from Italy by Fiocchi et al [44].

## Prophylaxis

We have observed mites in closed packages of wheat flour. Because the exposure to low temperatures inhibits mite proliferation and results in the transformation into protonymphal stages that are resistant to cold and do not reproduce actively, the recommendation has been made to store the flour in sealed containers in the refrigerator [9]. Kasti et al also suggested to store them in sealed glass or plastic containers [19].
